# Accuracy and stability of computer-aided customized lingual fixed retainer: a pilot study

**DOI:** 10.1186/s40510-022-00436-1

**Published:** 2022-11-21

**Authors:** Seung-Hyun Kang, Jae-Sung Kwon, Chooryung Judi Chung, Jung-Yul Cha, Kee-Joon Lee

**Affiliations:** 1 Department of Orthodontics, The Institute of Craniofacial Deformity, College of Dentistry, Yonsei University, 50-1 Yonsei-ro, Seodaemun-gu, Seoul, Korea; 2 Department and Research Institute of Dental Biomaterials and Bioengineering, Yonsei University College of Dentistry, Seoul, 03722 Korea; 3 Department of Orthodontics, Gangnam Severance Hospital, 211, Eonju-ro, Gangnam-gu, Seoul, Korea

**Keywords:** Lingual fixed retainer, Computer numerical control, Cutting, Bending, Thermocycling

## Abstract

**Background:**

With advances in digital technology, new types of lingual fixed retainers are being developed. However, there are few studies that quantitatively evaluate the accuracy and stability of lingual fixed retainers. The aim of this study was to assess the accuracy and stability of two types of computer-aided customized lingual fixed retainers and a conventional lingual fixed retainer.

**Methods:**

A total of 10 maxillary and 10 mandibular duplicated dental models were selected, and then, three types of retainers were fabricated on the canine-to-canine area for each model. To evaluate accuracy, wire clearance at interproximal area (WCI) was measured using superimposition analysis. Initial flatness deformation was also measured for vertical distortion of retainers. Lateral width, anteroposterior length, and flatness deformation were measured at three-time points for stability assessment. Thermocycling was used to induce 6 months of time flow.

**Results:**

The custom-bent group showed significantly higher WCI than the custom-cut and manual groups in the maxillary arch (*P* = 0.002). The custom-cut group showed significantly less flatness deformation, which was followed by the custom-bent and manual groups in both the maxillary and mandibular arch (*P* < 0.001). There was no significant difference in stability between the three retainer groups during 5100 cycles of thermocycling (corresponding to 6-month period).

**Conclusions:**

Since there was no difference in stability between the three groups, it is recommended to use custom-cut type retainers in light of accuracy. However, accuracy and stability are not the only factors to consider when selecting type of retainers. Because each retainer has advantages and disadvantages, the type of retainers should be decided in consideration of the clinical environment.

## Introduction

After completion of orthodontic treatment, teeth tend to return to their original positions [1], and Sadowsky and Sakols [2] reported that after 20 years of long-term follow-up, about 72% of 96 patients showed a tendency to relapse. Therefore, most patients use fixed or removable retainers to stabilize the occlusion after treatment [3]. The lingual fixed retainer is preferred over the removable retainer in that it is esthetically pleasing, does not require patient cooperation, and does not cause soft tissue discomfort or pronunciation problems [4]. In the case of labial orthodontic appliances, the use of straight wire was available with the development of a preadjusted appliance. In the lingual appliances, however, it is impossible to use a straight wire because the lingual surface of the tooth has a variety of shapes, greater curvature, and many anatomic variations compared to the labial surface [5]. Therefore, for fabrication of the conventional lingual fixed retainer, direct wire bending was essential to suit the individual shape of the teeth, and the multistranded wire retainer introduced by Zachrisson in 1982 has been considered the gold standard [6].

However, a disadvantage of the conventional lingual fixed retainer of the manual method is that it requires a lot of time to fabricate. To overcome this, a new type of lingual fixed retainer was developed with the advancement of digital technology [7]. Wire production using digital technology and three-dimensional scan models is based on computer numerical control (CNC). Cutting machines and bending machines are used as CNC machine tools to produce orthodontic wires. An example use of cutting machines is Memotain (CA-Digital, Hilden, Germany), a 0.014 × 0.014 inch lingual fixed retainer made by cutting a nickel–titanium metal plate [8, 9]. Examples of bending machine use include Suresmile (Dentsply Sirona, York, USA) and Incognito (3 M-Unitek, Monrovia, USA), in which the robot bends the wire to produce a customized wire [10, 11].

Recently, bending machines for the fabrication of lingual fixed retainers have been developed. According to previous studies, retainers fabricated using cutting machines have high positioning accuracy and excellent physical properties but have disadvantages in that they require a separate custom-made request, incur additional costs, and take several days to a week to deliver [12]. However, the bending machine for fabricating lingual fixed retainers has a relatively low price and is therefore accessible to the clinic. In addition, since it is possible to fabricate the retainer on the day of the visit, the number of visits of the patient is reduced.

Meanwhile, the accuracy and stability of the lingual fixed retainer are a clinically important issue. If the accuracy of the retainer is low, it is difficult to passively bond to the ideal position, and unwanted tooth movement may occur due to active force [13]. Furthermore, the gap between the retainer and the teeth widens, which increases patient discomfort and the possibility of occlusal interference [14]. Regarding the stability of the retainer, Aycan et al. [15] noted the malalignment of teeth and spacing between teeth due to wire deformation as disadvantages of the multistranded wire. Shaughnessy et al. [13] reported that when a multistranded wire is cut, untwisting occurs at the end of the wire due to internal tension, which can lead to torque discrepancy. Samson et al. [16] mentioned dimensional stability as a condition that fixed retainer wires should have.

Many studies have been conducted on the digitally fabricated lingual fixed retainers. Hu et al. [17] reported that the retainer fabricated using CAD/CAM is more efficient than the conventional retainer, requiring less production time, and is more stable because it is not affected by differences in the shape of teeth. Kravitz et al. [9] reported that the retainer fabricated using CAD/CAM was superior to the conventional retainer in terms of accuracy, prevention of occlusal interference, and corrosion resistance. As a result, digitally fabricated lingual fixed retainers are rapidly replacing the existing labor-dependent manual lingual fixed retainers.

However, there are few studies that quantitatively evaluate the accuracy of the retainer. The study by Wolf et al. [18], which performed quantitative measurements, has a limitation in that there is no control group setting. Similarly, studies evaluating the size stability of the retainer over time based on laboratory research have not been conducted. Additionally, previous studies did not include the recently introduced CNC bending type lingual fixed retainer. Therefore, this study aims to evaluate the accuracy and stability of two types of computer-aided customized lingual fixed retainers (CNC cutting and CNC bending) and a manually fabricated lingual fixed retainer. The null hypothesis of this study is that there is no significant difference in the accuracy and stability of three types of retainers.

## Materials and methods

### Samples

**Fig. 1:**
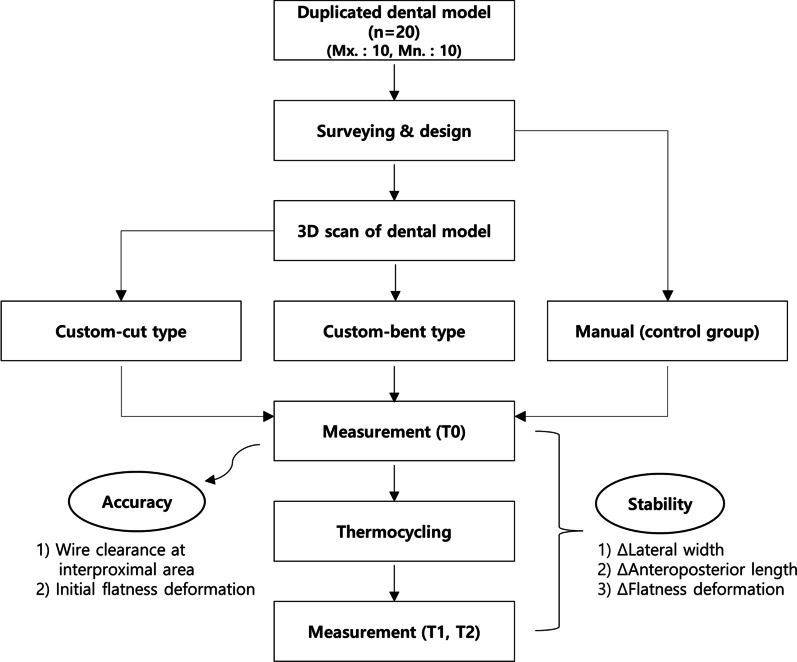
Flowchart of the study design

The sample consisted of 20 duplicated dental models (10 each for the maxillary and mandibular arches) of patients scheduled for debonding at the Department of Orthodontics, Yonsei University Dental Hospital after March 2021. Criteria for inclusion were as follows: (1) no missing teeth, prosthesis, malformed teeth, or spacing in the anterior area (2) clear shape of the interproximal area of the dental models. The range of the lingual fixed retainer was designated from the canine to the contralateral canine. According to the fabrication method, retainers were divided into custom-cut group (CNC cutting), custom-bent group (CNC bending), and manual group (control group). The overall scheme is shown as follows (Fig. 1).

### Retainer design

**Fig. 2:**
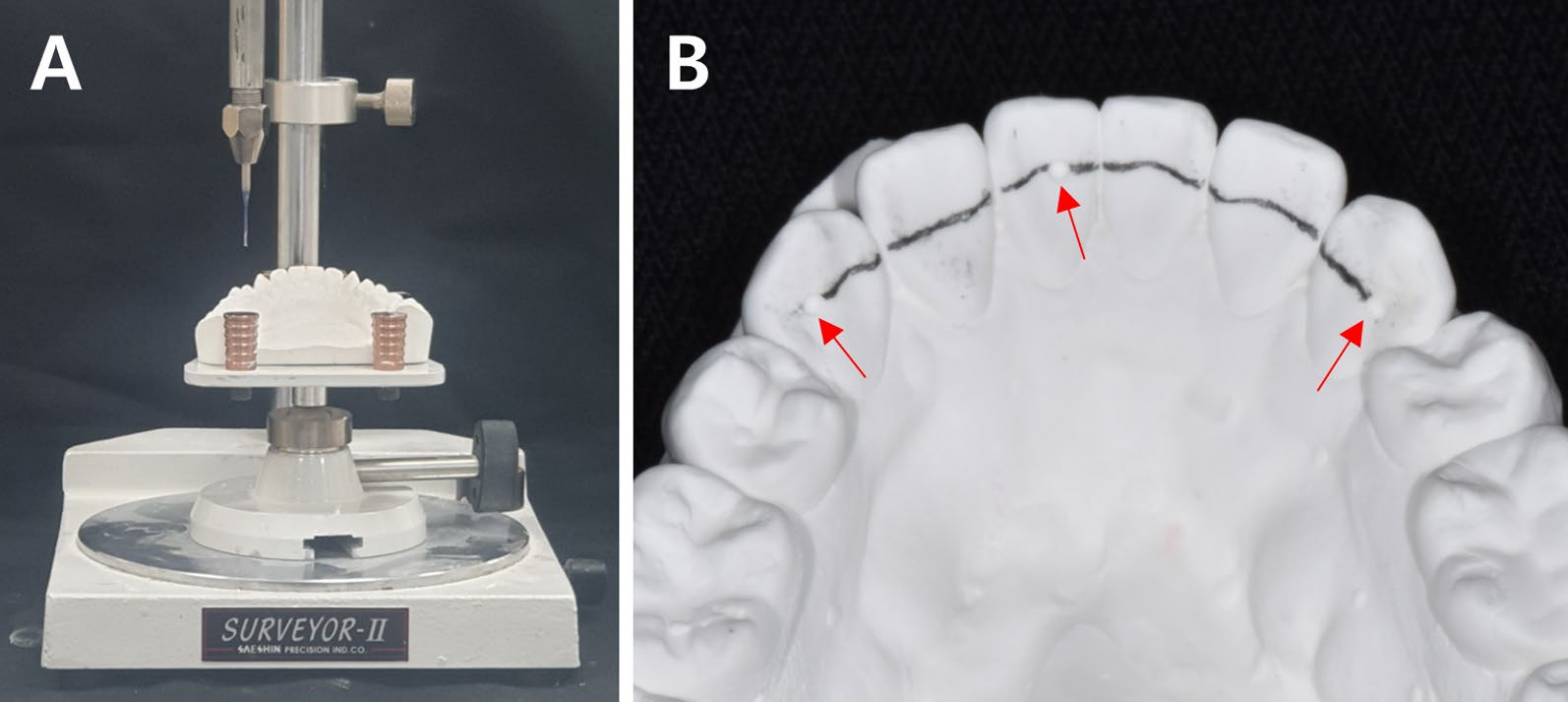
Design of lingual fixed retainer. **A** Design on one plane using dental surveyor. **B** After formation of small dimples (arrows) on two canines and one central incisor using round bur

A dental surveyor was used on the duplicated dental model to design the retainer from the canine to the contralateral canine in one plane. Then, in the maxillary arch, a small dimple was formed on the distal side of both canines and the center of the maxillary right central incisor using a round bur. In the mandibular arch, a dimple was formed on the distal side of both canines and the center of the mandibular left central incisor (Fig. 2).

### Retainer fabrication

#### Custom-cut group

The duplicated dental models were scanned, and stereolithography (STL) file was extracted to order the fabrication of Titainer (Lingualign Corp, Pohang, Korea), which is made of titanium alloy with a thickness of about 0.35 mm. It was requested to pass through three dimples located on one plane of the dental models but bypass the interior of the dimples. The fabricated retainer was cut at the mesial side of the dimple formed on both canines.

#### Custom-bent group

**Fig. 3:**
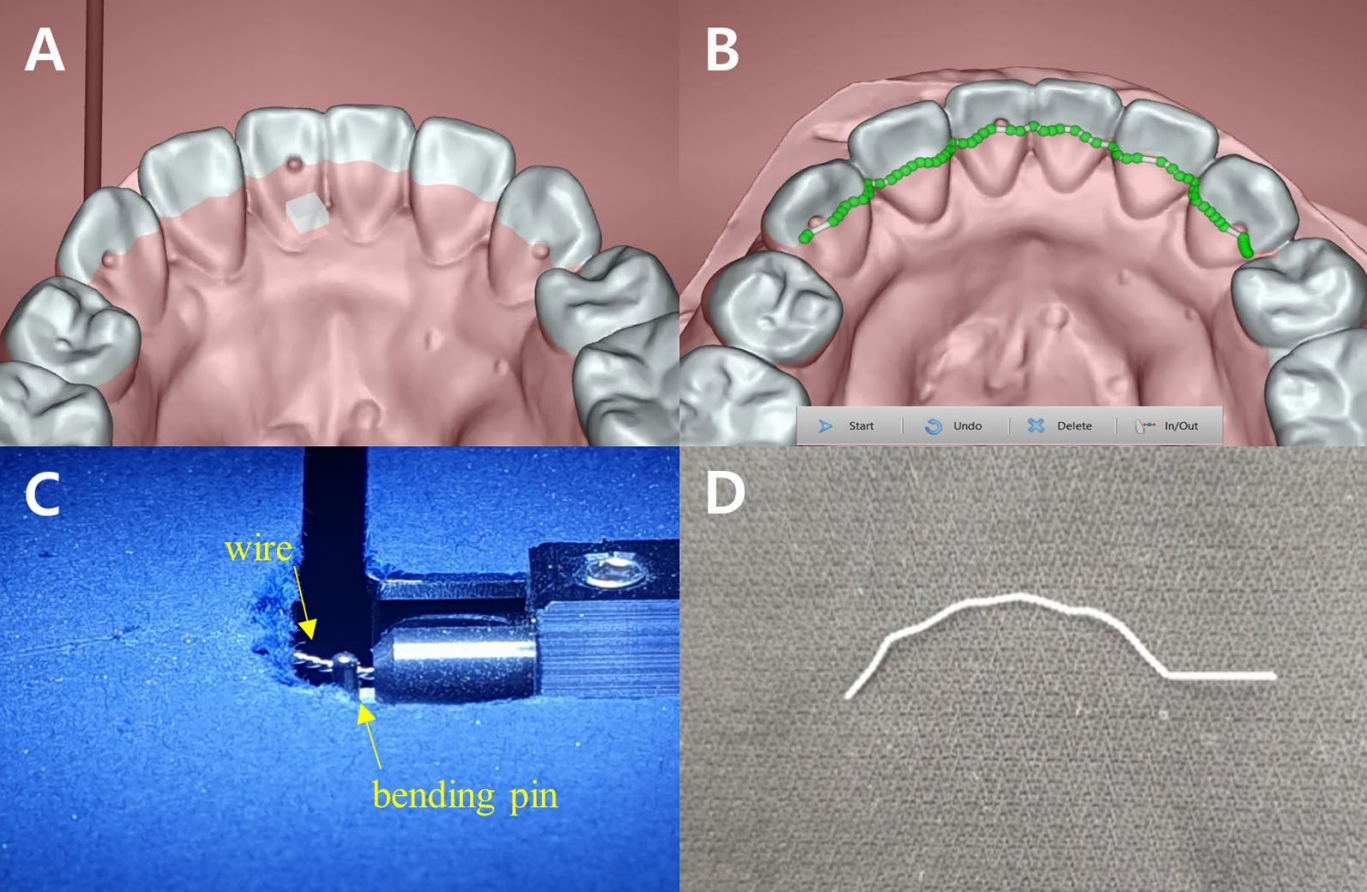
Fabrication of custom-bent type lingual fixed retainer. **A** Plane setting based on small dimples of dental model. **B** Design of retainer on reference plane (bypassed small dimples). **C** Bending process of wire. **D** Final product

Using the scan model and CAD software FixR (YOAT Corp., Lynnwood, USA), the retainer was designed to pass through three dimples of the dental models but bypass the interior of the dimples. Then, the retainer was fabricated through CAM software Bender1 (YOAT Corp., Lynnwood, USA) (Fig. 3). The wire used for fabrication was Dentaflex (Dentaurum GmbH & Co., Ispringen, Germany), a 0.5-mm-thick three-stranded stainless steel wire. The fabricated retainer was cut at the mesial side of the dimple formed on both canines.

#### Manual group

Dentaflex was used as in the custom-bent group, and one orthodontist bent the wire along the line designed with the dental surveyor. As with other types of retainers, the interior of the dimples was bypassed and the retainer was cut at the mesial side of the dimple formed on both canines.

### Measurement

#### Accuracy

##### Wire clearance at interproximal area (WCI)

**Fig. 4:**
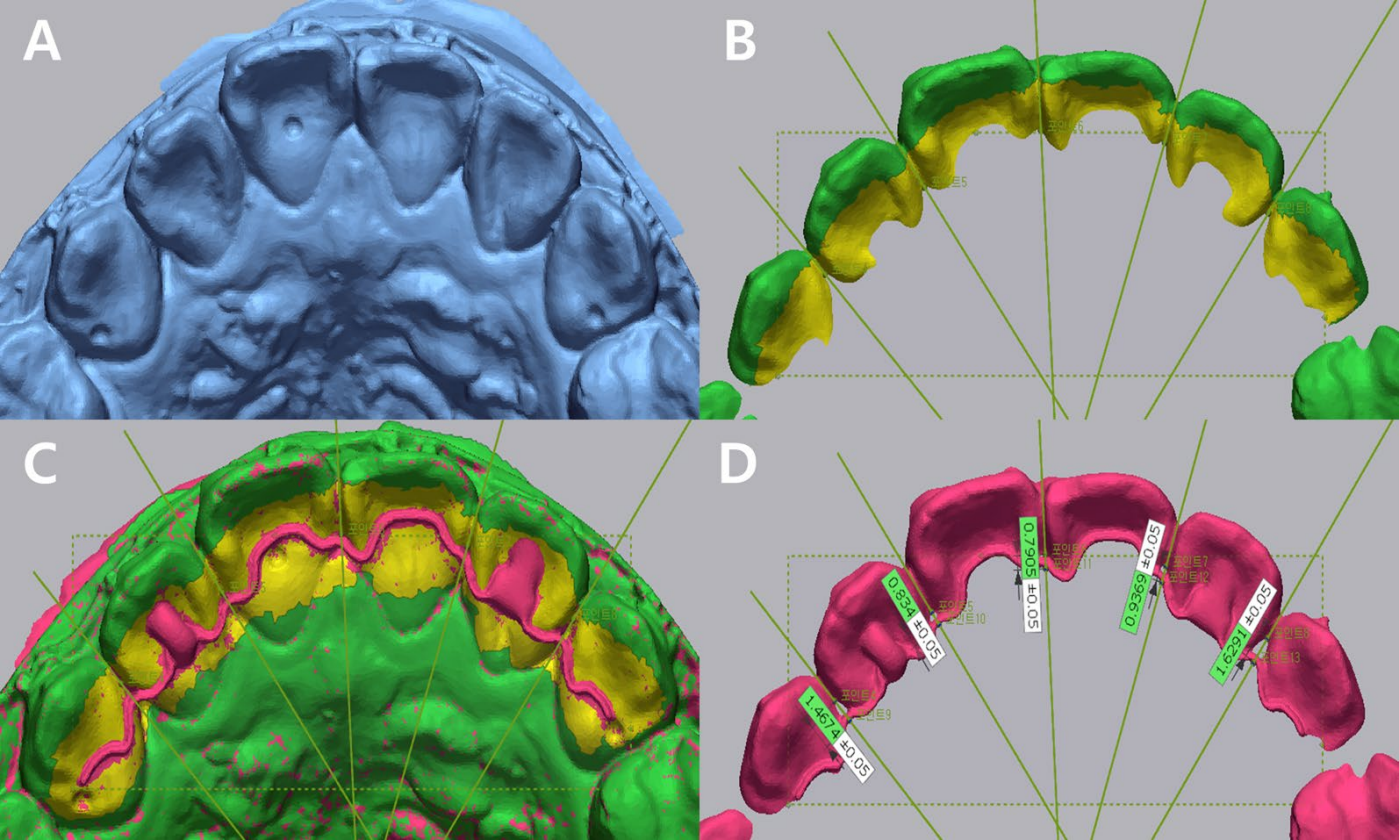
Measurement of WCI. **A** Initial scan model with three dimples. **B** Setting of reference plane (dotted line), five interproximal surfaces (solid line) and five reference points. Yellow area indicates the part excluded when superimposing. **C** After superimposition between the initial scan model and the model with the retainer attached. **D** Length measurement between two points in five interproximal areas

Geomagic Control X (3D systems, Rock Hill, USA) software was used to set the reference plane using the dimples in the initial scan model [19, 20]. Then, five interproximal surfaces were set from the canine to the contralateral canine. Among the line of intersection of the reference plane and each interproximal surface, a point on the tooth surface was selected as the reference point for measurement. After applying the scan spray to the fabricated retainer, it was fixed to the dental model with wax and scanned. Then, the initial scan model with the reference point set and the scan model with the retainer attached were superimposed by best-fit method. After measuring the distance from the reference point to the most palatal or lingual border of the retainer, the average value of measurements in five interproximal surfaces was calculated (Fig. 4). The value obtained by subtracting the average thickness of the retainer (0.35 mm for the custom-cut group, 0.5 mm for the custom-bent and manual group) from this average value was defined as WCI. It was considered that the smaller WCI, the higher the accuracy of the retainer.

##### Initial flatness deformation

**Fig. 5:**
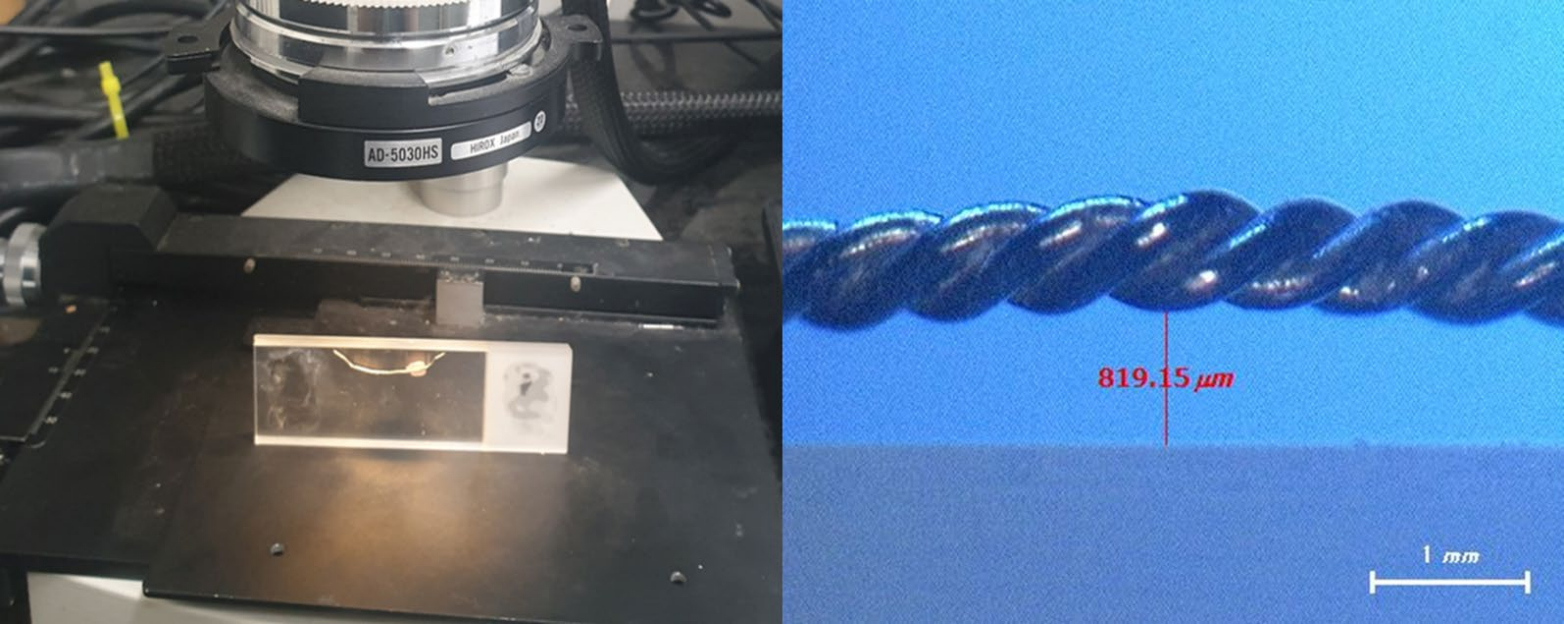
Measurement of flatness deformation (70 × magnification)

After fixing the retainer to the slide glass with wax, the farthest vertical distance from the slide glass to the retainer was defined as flatness deformation (FD). In the case of a multistranded wire, the most convex point of the wire was taken as the reference. After magnifying 70 times using an Image analyzer (Hyrox Korea Co, Anyang, Korea), the distance was measured on Image partner 4.0 (Saramsoft Co., Anyang, Korea) software (Fig. 5). The initial flatness deformation was the flatness deformation measured at T0 (immediately after the fabrication of the retainer), and it was determined that the smaller this value, the higher the accuracy of the retainer.

#### Stability

**Fig. 6:**
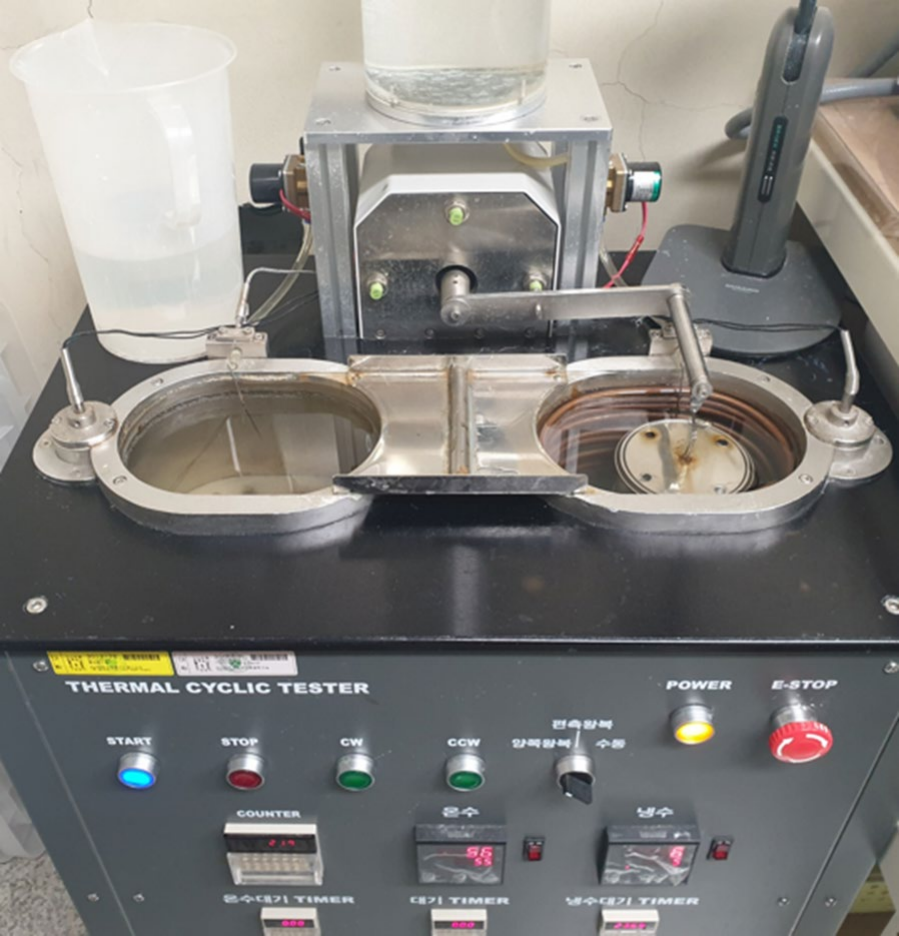
Thermal cyclic tester (R&B Inc., Daejeon, Korea)

To evaluate the three-dimensional size stability of the retainer, lateral width (LW), anteroposterior length (AP), and FD were measured at three-time points: T0, T1 (850 cycles of thermocycling for simulation of 1 month), and T2 (5100 cycles for 6 months). It was determined that the smaller the absolute values of the changes in these values with time (ΔLW, ΔAP, ΔFD), the higher the stability of the retainer. In this study, thermocycling was used to induce 6 months of time flow under oral temperature conditions (37 ℃) (Fig. 6). After immersing the retainer sample in physiological saline, the temperature of the cold bath and warm bath was set to 5 ℃ and 55 ℃, respectively. Dwell time and transfer time were set to 45 s and 5 s, respectively [21]. Because it is known that 10,000 cycles of thermocycling approximately correspond to oral conditions at 37 ℃ for one year, 850 and 5100 cycles were determined as values corresponding to 1 month and 6 months, respectively [22, 23].

##### LW

**Fig. 7:**
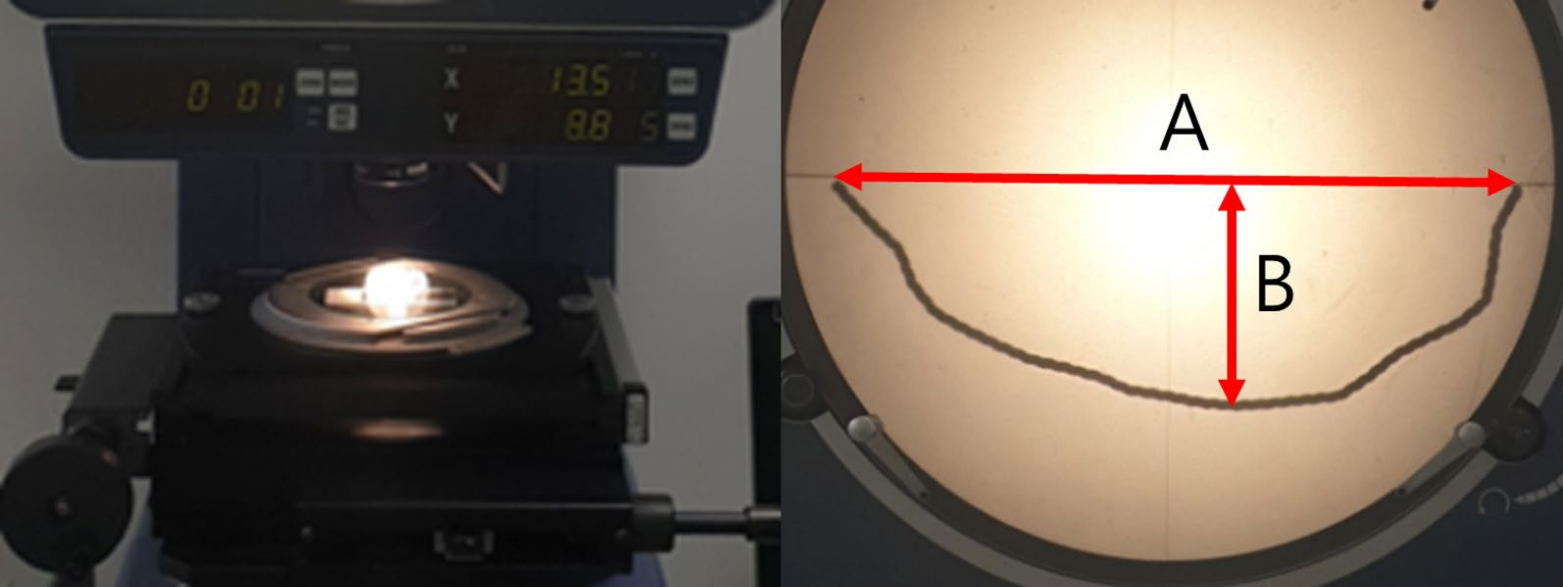
Measurement of LW and AP using profile projector. **A** Lateral width. **B** Anteroposterior length

After placing the retainer on a flat plane, the distance between the centers of both ends of the retainer was defined as LW. A profile projector (Mitutoyo, Kawasaki, Japan) was used for the measurement (Fig. 7).

##### AP

After placing the retainer on a flat plane, the anteroposterior distance farthest from the straight line connecting the centers of both ends of the retainer to the inner border of the retainer was defined as AP. A profile projector was used for the measurement (Fig. 7).

### Statistical analysis

SPSS software version 25.0 (IBM Corp., Armonk, USA) was used for statistical analysis of data. For reliability analysis, intraclass correlation coefficient (ICC) was calculated. One week after the first measurement, one researcher randomly selected 30% of the total samples and performed the second measurement. ICC values showed 0.88 or higher for all measurements.

For normality test, the Shapiro–Wilk test was applied. One-way ANOVA was used to compare WCI and initial flatness deformation in the maxillary arch and initial flatness deformation in the mandibular arch, followed by Tukey’s post hoc test for multiple comparisons. The Kruskal–Wallis test was used to compare WCI in the mandibular arch, ΔLW, ΔAP and ΔFD between groups. When the *p* value was 0.05 or less, it was determined that there was a statistically significant difference.

## Results

### Accuracy

**Fig. 8:**
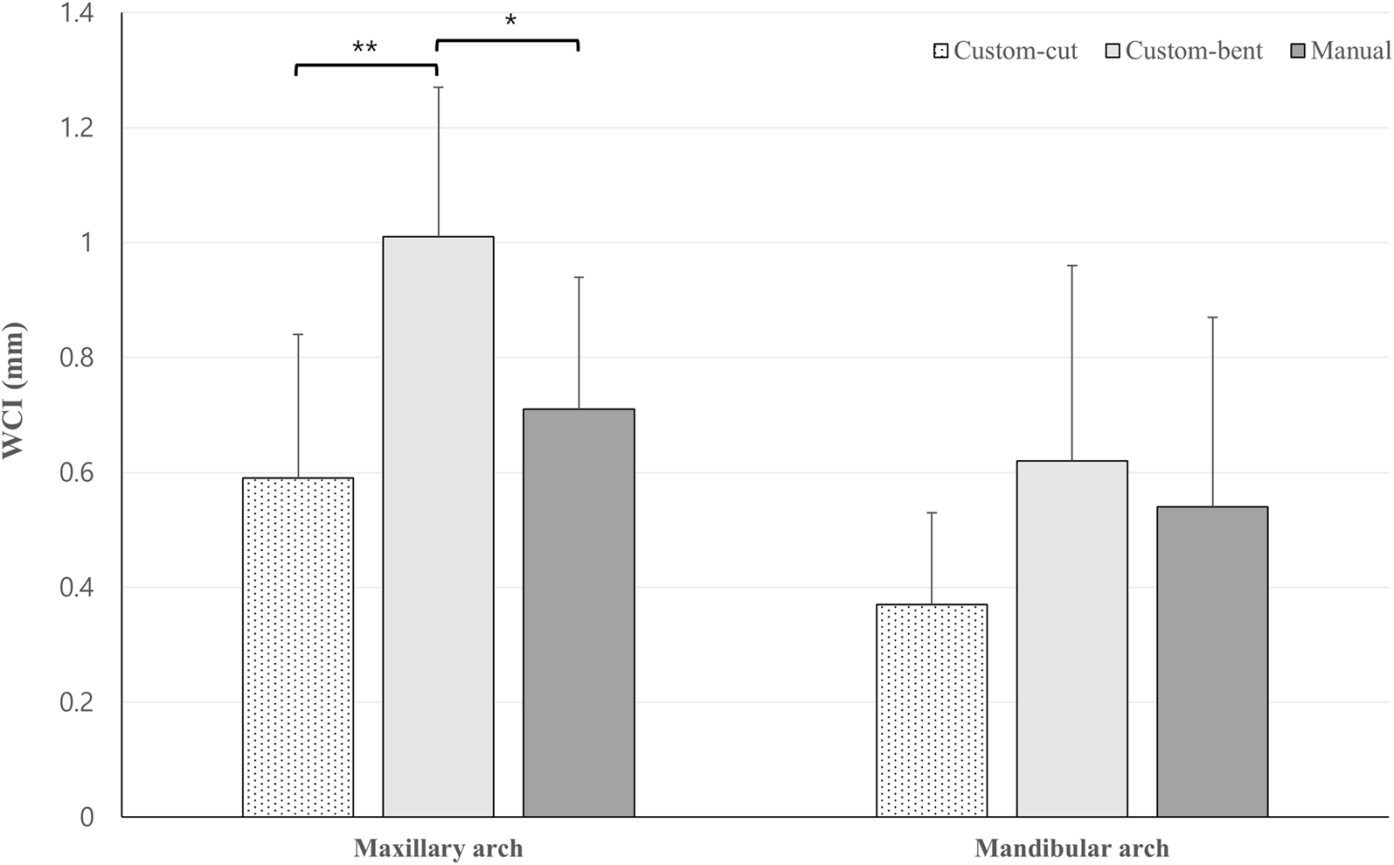
Comparison of WCI between three groups in maxillary and mandibular arch. *significance at the 0.05 level. **significance at the 0.01 level

Based on one-way ANOVA, there was a significant difference in WCI between the three groups in the maxillary arch (*p* = 0.002). As a result of post hoc test, the custom-bent group (1.01 ± 0.26 mm) showed significantly higher WCI than the custom-cut (0.59 ± 0.25 mm) and manual group (0.71 ± 0.23 mm). On the other hand, there was no significant difference between the three groups in the mandibular arch (*p* = 0.104) (Fig. 8).

**Fig. 9:**
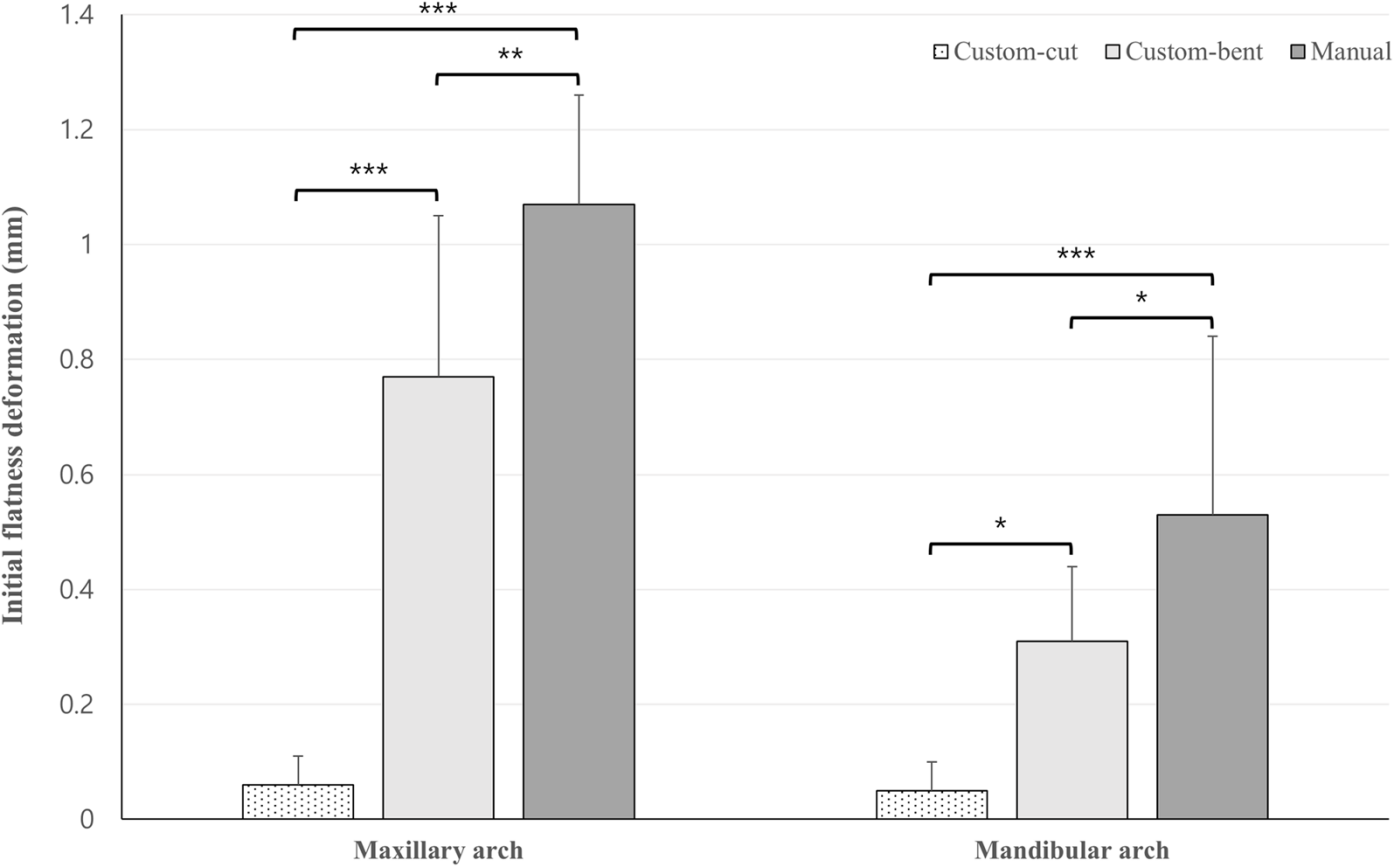
Comparison of initial flatness deformation between three groups in maxillary and mandibular arch. *significance at the 0.05 level. **significance at the 0.01 level. ***significance at the 0.001 level

For initial flatness deformation, there was a significant difference between the three groups in the maxillary arch (*p* < 0.001). According to the result of multiple comparisons, the custom-cut group (0.06 ± 0.05 mm) showed significantly less flatness deformation, which was followed by the custom-bent (0.77 ± 0.28 mm) and manual group (1.07 ± 0.19 mm). As in the maxillary arch, there was a significant difference in initial flatness deformation in the mandibular arch (*p* < 0.001). The post hoc test also showed the same results as for the maxillary arch in the order of custom-cut (0.05 ± 0.05 mm), custom-bent (0.31 ± 0.13 mm) and manual group (0.53 ± 0.31 mm) (Fig. 9).

### Stability

The Kruskal–Wallis test showed that there were no significant differences in ΔLW, ΔAP, ΔFD values between three groups in T0-T1 (thermocycling 850 cycles) and T0-T2 (thermocycling 5100 cycles) (Table 1).

**Table 1:** Comparison of ΔLW, ΔAP, ΔFD in three retainer groups

Variable	Mean ± SD (mm)			*p*-Value
	Median (IQR) (mm)			
	Custom-cut	Custom-bent	Manual	
ΔLW (T0-T1)	0.04 ± 0.03	0.06 ± 0.05	0.06 ± 0.04	0.207
	0.03 (0.01–0.05)	0.05 (0.02–0.07)	0.05 (0.02–0.08)	
ΔLW (T0-T2)	0.03 ± 0.03	0.08 ± 0.08	0.06 ± 0.06	0.092
	0.03 (0.01–0.05)	0.07 (0.01–0.15)	0.05 (0.02–0.11)	
ΔAP (T0-T1)	0.03 ± 0.02	0.03 ± 0.03	0.04 ± 0.03	0.203
	0.02 (0.01–0.04)	0.02 (0.01–0.05)	0.03 (0.02–0.07)	
ΔAP (T0-T2)	0.03 ± 0.03	0.04 ± 0.04	0.03 ± 0.02	0.383
	0.02 (0.01–0.05)	0.03 (0.02–0.05)	0.03 (0.02–0.04)	
ΔFD (T0-T1)	0.04 ± 0.06	0.05 ± 0.05	0.04 ± 0.03	0.215
	0.02 (0.00–0.04)	0.04 (0.01–0.08)	0.05 (0.01–0.07)	
ΔFD (T0-T2)	0.04 ± 0.04	0.04 ± 0.03	0.06 ± 0.05	0.402
	0.04 (0.01–0.07)	0.02 (0.01–0.05)	0.05 (0.02–0.10)	

SD: standard deviation; IQR: interquartile range.

P values were calculated using Kruskal–Wallis test (α = 0.05).

## Discussion

After the completion of orthodontic treatment, the tendency of relapse, including the decrease in intercanine width, is clear [24]. For this reason, most orthodontists prefer permanent retention using lingual fixed retainers [25]. With the advancement of digital technology, new types of lingual fixed retainers are being developed and studies for comparison between conventional and new types of lingual fixed retainers are actively being conducted. Nevertheless, evidence-based data available for clinical use are still insufficient and this makes it difficult for clinicians to determine which type of lingual fixed retainer is appropriate for each patient. In particular, there are few studies directly comparing the CNC cutting type retainer with the recently introduced CNC bending type retainer. In addition, based on the literature review prior to this study, there was no study examining the size stability of lingual fixed retainer over time. As mentioned previously, the accuracy and stability of the retainer are very important factors from a clinical standpoint. Therefore, this study evaluated potential differences in accuracy and stability between the retainers including CNC bending type.

Most of the previous studies evaluating the performance of lingual fixed retainers have been clinical studies. However, in this way, it is impossible to compare different types of retainers for the same patient. In addition, this study design presents a challenge in that it is difficult to control variables such as individual occlusal force or tongue pressure when evaluating stability. Therefore, this study was conducted through in vitro, laboratory research to purely compare the effect of the wire. Additionally, thermocycling was used to induce long-term time flow under oral temperature conditions during stability evaluation. Kartal and Kaya [8] reported that most retainer failures occurred within 6 months, which is considered a clinically significant observation period and was determined as the time standard for stability evaluation.

Regarding the accuracy of the retainer, Wolf et al. [18] measured three-dimensional position changes in the interproximal area by superimposing the scan data before and after attachment of the CNC cutting type retainer. A position change of 0.5 mm was considered clinically significant, and as a result, a displacement of less than 0.5 mm was observed in all measurements. Kravitz et al. [9] reported that the better the interproximal fit, the higher the accuracy of retainer. Therefore, in this study, WCI was selected as a measurement for accuracy evaluation. Since the gap between the retainer and the tooth surface is filled with resin, the area where the clinician is sensitive to the accuracy is highly likely to be the interproximal area. Thus, it is reasonable to use WCI as a criterion for evaluating the accuracy of the lingual fixed retainer.

**Fig. 10:**
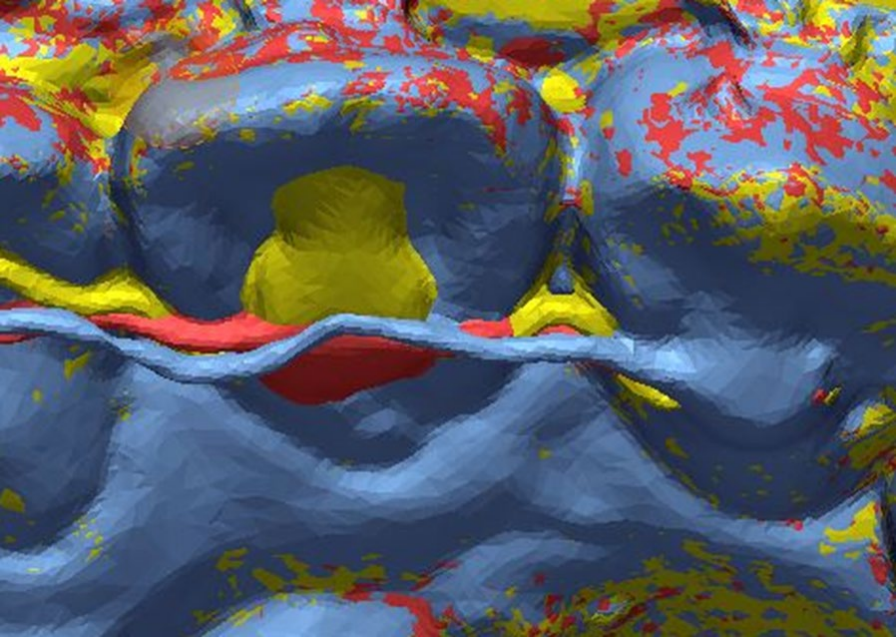
Superimposition of three types of retainers simultaneously (yellow: custom-cut group, blue: custom-bent group, red: manual group)

According to our findings, WCI of the custom-bent group was significantly higher than that of the custom-cut and manual groups in the maxillary arch (Fig. 10). The manual group showed a higher WCI than the custom-cut group, but there was no statistical significance. This result seems to be due to differences in the characteristics of the fabricating methods. In the CNC cutting type retainer, a high level of interproximal extension is possible because it is fabricated by cutting a metal plate. In the case of CNC bending type retainer, there is a limit to the curvature that can be given to the retainer due to the thickness of the bending pin itself (Fig. 3-C). Therefore, it becomes difficult to reproduce the shape in a narrow area less than the bending pin thickness.

Unlike the maxillary arch, there was no significant difference in WCI between the three types of retainers in the mandibular arch, which may be due to the difference in anatomic structure between the maxillary and mandibular teeth. Kravitz et al. [9] reported that the CNC cutting type retainer has an advantage compared to conventional retainers, especially in the maxillary arch. The study reported that it was difficult to closely fit the retainer to the maxillary teeth with the conventional retainer because of the prominent marginal ridge of the maxillary teeth or the unusual tooth shape. In this study, since samples with malformed teeth in the anterior area were excluded, the main reason for the different WCI values is thought to be the difference in marginal ridge prominence and interproximal shape. Because the mandibular teeth have a smaller curvature than maxillary teeth, it can be predicted that bending pins or human hands will be able to implement an anatomic shape similar to that of a CNC cutting type retainer.

Initial flatness deformation is another measurement related to accuracy in this study. The larger the value, the greater the vertical displacement of the retainer from the initially set reference plane. There was a significant difference in the initial flatness deformation between three groups in both the maxillary and mandibular arch. Manual, custom-bent, and custom-cut groups showed the highest values in the order, which seems to be related to the characteristics of the fabrication method. Because the manual method applies bending many times by hand, it is difficult to control the plane distortion during each bending. Furthermore, since it is difficult to monitor fine plane distortion with the naked eye, it is challenging to precisely maintain the plane flatness during fabrication. The CNC bending method lowers the chance of plane distortion because the number of bendings is less than that of the manual method. However, the aspect of plane distortion varies depending on whether the bending pin bends the concave or convex part of the multistranded wire. Therefore, if a single-stranded wire is used, the initial flatness deformation is expected to decrease. However, in this case, other problems such as a decrease in the wire flexibility may occur.

Some previous studies have questioned the stability of conventional lingual fixed retainers. Kartal et al. [7] reported a possibility of tooth movement due to distortion of the multistranded wire. Pazera et al. [26] reported that further research is needed on the biomechanical properties of multistranded wire in relation to the complications of lingual fixed retainers. Based on our findings, no statistical significance was observed in any measurement related to stability. That is, during the 5100 cycles of thermocycling, the difference in stability was not significant and the custom-bent and manual groups using multistranded wire were not inferior to the custom-cut group. As a result, the fabrication method and wire material of the retainers used in this study were different, but it is not expected that these factors contribute to the stability of the retainers. However, further studies are needed to evaluate long-term stability.

This study evaluated the accuracy and stability of two types of computer-aided customized lingual fixed retainers and conventional retainers through laboratory experiment. There was no difference in stability during 5100 cycles of thermocycling, and it was confirmed that there was a difference in accuracy depending on the type of retainer. It means that it is clinically simple to attach to the ideal position when using the CNC cutting type retainer, which is more accurate and can reduce chair time and the possibility of complications. On the other hand, the accuracy of the CNC bending type retainer is somewhat lower, but it is fabricated in the shortest time and can be attached quickly on the same day the patient visits the hospital. Compared to the manual retainer, the CNC bending type retainer has an advantage in that the initial flatness deformation is lower, because the minimum number of bendings is given at a predetermined point. It is speculated that this will result in less wire deformation and stronger resistance to wire fracture due to fatigue. However, to confirm this finding and acquire more scientific data, further studies must be conducted. In the mandibular arch, the risk of occlusal interference is low, and relatively high accuracy can be expected in terms of tooth shape, so it is worth considering the use of a CNC bending type retainer.

There are some limitations in this study. First, there are factors that can lower the accuracy of measurement. In the case of CNC cutting type retainer in particular, an error may occur in setting the reference plane because fabrication is outsourced. Second, since thermocycling was used for stability evaluation in this study, there is an error from the actual period and caution is required when interpreting the results. Lastly, only the canine-to-canine lingual fixed retainer was evaluated. Further studies should evaluate its stability over a long period of more than 6 months. In addition, since the premolar-to-premolar retainer is widely used in clinical practice, it is expected that expanding the sample to the premolar-to-premolar retainer will yield more information for clinicians.

## Conclusions

In this study, the accuracy and stability of three types of lingual fixed retainers were compared. The conclusions obtained in this study were summarized as follows:

- Based on WCI, the custom-cut and manual groups showed higher accuracy than the custom-bent group in the maxillary arch, but there was no significant difference between the groups in mandible.
- The custom-cut group showed significantly less flatness deformation, which was followed by the custom-bent and manual group in both the maxillary and mandibular arch.
- There was no significant difference in stability between the three groups during 5100 cycles of thermocycling (corresponding to 6-month period).

Since there was no difference in stability between the three groups, it is recommended to use custom-cut type retainers in light of accuracy. However, accuracy and stability are not the only factors to consider when selecting the type of retainers, and since each retainer has advantages and disadvantages, the type of retainers should be decided with consideration of the clinical environment.

## Data Availability

The datasets used and/or analyzed during the current study are available from the corresponding author on reasonable request.

## Abbreviations

CNC: Computer numerical control
CAD: Computer-aided design
CAM: Computer-aided manufacturing
STL: Stereolithography
WCI: Wire clearance at interproximal area
FD: Flatness deformation
LW: Lateral width
AP: Anteroposterior length
